# Antimicrobial Stewardship in oral and maxillofacial surgery: melting the iceberg

**DOI:** 10.1017/ash.2026.10370

**Published:** 2026-05-11

**Authors:** Insa Joost, Melanie Kempe, Barbara Eirmbter, Colin R. MacKenzie, Lara Schorn

**Affiliations:** 1 Institute of Medical Microbiology and Hospital Hygiene, University Hospital Duesseldorf, Duesseldorf, Germany; 2 Department of Pharmacy, HELIOS Hospital Schwelm: HELIOS Klinikum Schwelm, Germany; 3 Department of Pharmacy, University Hospital Duesseldorf, Germany; 4 University Hospital Duesseldorf, Germany; 5 Department of Oral and Maxillofacial Surgery, University Hospital Duesseldorf, Germany

## Abstract

**Objective:**

Irrational antibiotic consumption contributes to antimicrobial resistance, adverse effects, and rising costs. Antimicrobial Stewardship (AMS) supports rational antibiotic use through restrictive and supportive measures. In oral and maxillofacial surgery (OMFS), antibiotic consumption is consistently high, and evidence-based guidelines are often lacking. We evaluated a multimodal AMS intervention on antibiotic use in an OMFS department.

**Design:**

Before–after study.

**Setting:**

OMFS department of a German university hospital.

**Patients:**

Hospitalized patients treated in the OMFS department.

**Intervention:**

For 12 months, the AMS team conducted weekly ward visits, providing recommendations regarding antibiotic therapy and surgical antibiotic prophylaxis (SAP). In parallel, a mandatory consensus guideline covering these aspects was developed and implemented. Antibiotic use, prevalence, duration of therapy, and SAP before, during, and after the intervention were evaluated; clinical outcomes were not assessed.

**Results:**

Pre-intervention, over 70% of patients received antibiotics. Median duration of antibiotic therapy and SAP was 12 and 9 days, respectively. Antibiotic consumption reached 63.8 defined daily dosages per 100 patient days, exceeding national values (median 40.1). During the intervention, antibiotic prevalence decreased below 50% and the duration of antibiotic therapy and SAP was shortened to 9 and 3 days, respectively. Antibiotic consumption decreased by 26% whereas national values increased by nearly 50%. After weekly consultations ceased, antibiotic use rose again but remained below national levels.

**Conclusions:**

Antibiotic consumption is high in OMFS. Regular AMS support including consensus guidelines can reduce antibiotic use substantially and sustainably. However, clinical outcomes need to be correlated and adequately staffed AMS teams are needed.

## Introduction

Several infectious disorders in oral and maxillofacial surgery (OMFS) require antibiotic therapy and perioperative surgical antibiotic prophylaxis (SAP) is routinely given to prevent surgical site infection for numerous interventions.^1–3^ Robust evidence regarding optimal choice and duration of antibiotic therapy is lacking. Furthermore, in an attempt to prevent infectious complications, prolonged SAP is often administered, especially in high-risk constellations like large tumor operations involving advanced flap techniques.^4,5^ For the majority of infections and interventions, no national or international guidelines exist regarding the choice of antibiotic, duration of therapy or length of antibiotic prophylaxis. In an attempt to “shield” the patient from possible infectious complications, this often leads to an overcautiously broad indication for antibiotic therapy and prolonged SAP.^6^ Excessive antibiotic consumption, however, is a known major health problem leading to increased side effects for patients, prolonged hospital stays, nosocomial infections with *C. difficile*, MRSA or VRE, increasing resistance and costs.^7,8^ Antimicrobial Stewardship (AMS) programmes aim to reduce unnecessary antibiotic therapy as well as prolonged antibiotic prophylaxis by different approaches. Audit and feedback modules as well as consensual guideline development and implementation have been proven to be effective in the reduction of antibiotic consumption.^9^

## Materials and methods

### Antibiotic consumption

Yearly antibiotic consumption was calculated as defined daily doses (DDD) per 100 patient days, in accordance with the WHO ATC/DDD methodology.^10^ Data collection was limited to antibiotics administered intravenously. Consumption data were extracted via the webKESS AVS (antibiotic consumption surveillance) module provided by the Robert Koch Institute (RKI), using routine hospital pharmacy dispensing data. Patient-day denominators were obtained from institutional administrative records. Data on perioperative single shot prophylaxis that had been administered outside the ward were not included. National average values were also provided by the RKI and consisted of the median antibiotic consumption calculated from data of a reference group of OMFS departments from German university hospitals. The number of comparator hospitals rose from 7 hospitals in 2017 to 13 in 2023 as more hospitals participated. Data were collected from 01.01.2017–31.12.2023.

### Study patients and data collection

**Figure 1.**
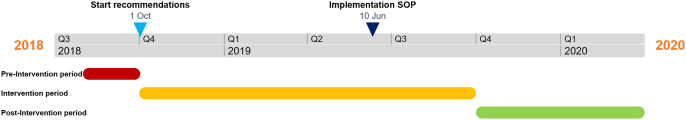
Time line of preintervention, intervention and postintervention periods.

The study was conducted at the university clinic Düsseldorf, Germany. The OMFS Department consists of an outpatient clinic and a 32-bed ward. Time line see Figure 1.

Preintervention (01.08.2018–30.09.2018): The AMS team consisting of a physician and a pharmacist, performed weekly visits on the OMFS ward over a period of eight weeks. All hospitalized patients were documented. Recorded data included main diagnosis, date and type of operation, microbiological results, antibiotic agent, dosage, route of administration, start, end, duration and indication (therapy or prophylaxis) of antibiotic therapy. No recommendations were given at this point.

Intervention (1.10.2018–30.9.2019): Over 12 months the weekly AMS visits were continued. Each patient receiving an antibiotic was discussed with the attending physician and written recommendations were given: Treatment adjustment (de-/escalation, cessation, scheduled stop, drug switch, change of doseor route) and recommendations for further diagnostics. Identical data as mentioned above were collected. In parallel, a literature review was conducted and a local consensus guideline was developed, which was officially implemented in June 2019, 8 months after the intervention started and mandatory for all OMFS physicians. It included recommendations for microbiological sampling as well as the antibiotic therapy of typical OMFS infectious disorders. Furthermore, it addressed perioperative antibiotic prophylaxis for all surgical procedures including recommendations on type, dose, route, duration, alternatives for penicillin-allergic patients as well as endocarditis prophylaxis.

Postintervention (1.10.2019–31.3.2020): After 12 months of weekly visits, the regular support was stopped. Over a postinterventional period of 6 months, irregular, unannounced follow-up visits were conducted.

### Statistics

Over the whole study period, 1,124 patient contacts were recorded. All hospitalized patients on the ward at the time of each weekly visit were assessed, so individual patients could be captured multiple times if present during multiple weeks. These data were pooled across all weekly visits within each calendar month to calculate the monthly point prevalence of antibiotic use, defined as the total number of patients receiving antibiotics divided by the total number of patients assessed. This pooled approach accounts for variation in weekly ward census. In addition, each antibiotic prescription was classified as either treatment or prophylaxis, based on clinical documentation. For the analysis of treatment and prophylaxis duration, duplicate patient entries were removed, resulting in a cohort of 551 unique patients evaluated for this part of the study. Single shot SAP that was administered outside the ward was not recorded.

Descriptive statistics were used to summarize the data. Categorical variables were reported as absolute numbers and percentages and continuous variables as medians with ranges. No inferential statistical tests were performed. All analyses were conducted using Microsoft Excel LTSC 2021, Version 16.0 (Microsoft Corp., Redmond, WA, USA).

### Ethics approval

The study was approved by the university’s ethic committee (study-no.: 2019-803).

## Results

### Antibiotic consumption before, during, and after the AMS intervention

**Figure 2.**
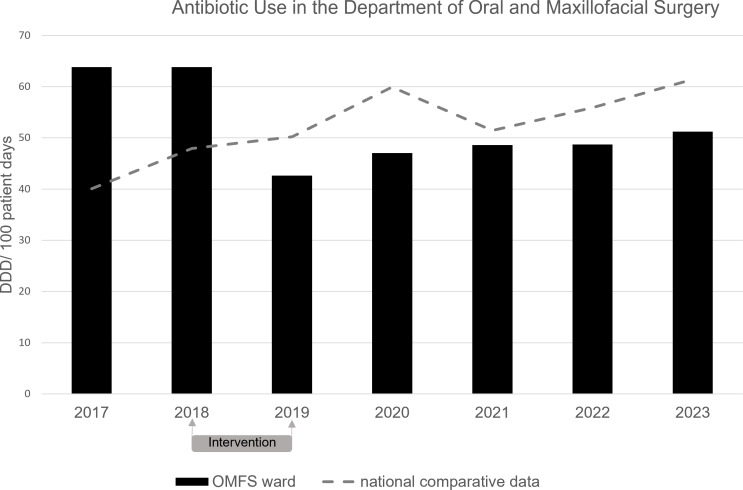
Antibiotic consumption in the department of oral and maxillifacial surgery (OMFS) from 2016 to 2023 in defined daily dosage (DDD)/ 100 patient days. Black bars represent the antibiotic consumption of the OMFS department at the University Hospital Duesseldorf. The dotted line represents the national average data (see material and methods).

Figure 2 illustrates antibiotic consumption before, during, and after the AMS intervention.

In 2017, antibiotic use in the OMFS department was 63.8 DDD per 100 patient-days, which was 59.1% above the national average (40.1 DDD per 100 patient-days). For 2018, when the intervention started in October, consumption at the OMFS department was still 33% above the nation average (63.8 and 47.9 DDD per 100 patient-days for OMFS and national average, respectively). The AMS intervention lasted for 12 months from October 2018 to October 2019. In 2019, antibiotic consumption in the OMFS department decreased to 42.65 DDD per 100 patient-days, compared to a national average of 50.2 DDD. In the postintervention period in 2020, the antibiotic consumption at the OMFS department rose to 47.3 DDD per 100 patient-days, however, the national average rose at a higher rate to 59.9 DDD per 100 patient-days.

This corresponds to a 26.3% reduction between the pre and postintervention period at the OMFS department whereas during the same time period an average increase of 49% in the comparator hospitals was seen.

This reduction persisted beyond the intervention period, which ended on September 30, 2019. For 2021 to 2023, the antibiotic consumption rose again but a) remained lower than preintervention levels and b) below the national average despite the cessation of the AMS intervention (Figure 2).

### Prevalence of antibiotic usage before, during, and after the AMS intervention

**Figure 3.**
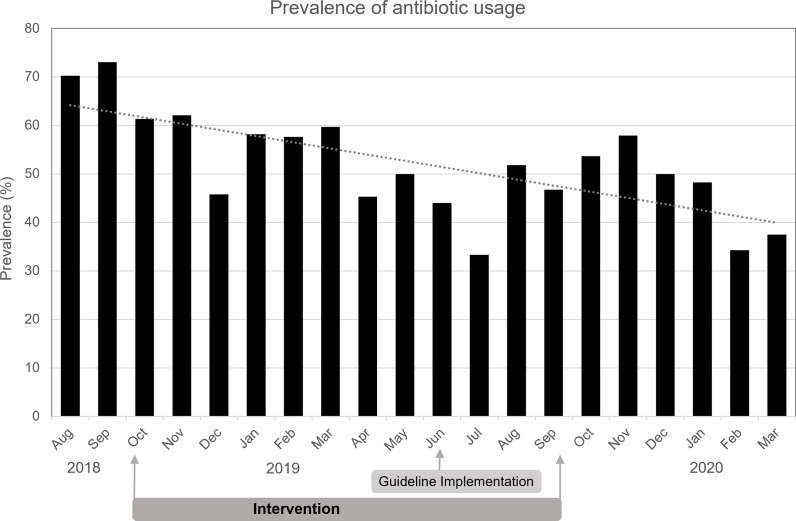
Antibiotic prevalence in the department of oral and maxillifacial surgery from august, 2018 till march, 2020. Black bars represent the antibiotic prevalence, the dotted line represents the linear trend line.

In August and September before the intervention, 167 patients were evaluated during weekly visits. 121 patients received an antibiotic, which reflects a prevalence in the preintervention period of 72%. With the start of the intervention—which consisted of continuous weekly visits, now including discussion of every patient with the ward team and the provision of written recommendations—the prevalence dropped to its lowest value of 33% in July 2019, shortly after the official introduction of the consensus guideline (Figure 3). Initially, the rate rose after cessation of the weekly visits and showed variability from month to month but remained below preintervention values throughout the whole postintervention period. Notably, no upward trend in antibiotic prevalence with increasing time since the end of the intervention period was observed.

### Surgical antibiotic prophylaxis (SAP) and antibiotic therapy

**Figure 4.**
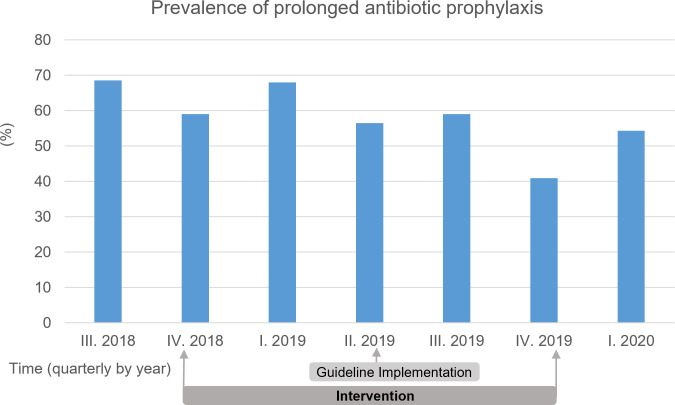
Prevalence of prolonged surgical antibiotic prophylaxis (SAP) at the department of oral and maxillofacial surgery in the preintervention, intervention and postintervention period.

At each visit, the usage of antibiotics was classified as being either prophylactic or therapeutical. In the preintervention period, 68.6% of all patients receiving antibiotics on the ward, as prolonged (ie, more than single shot) SAP. During the intervention phase, this figure fluctuated considerably; however, over the entire period, a reduction in the prevalence of prolonged SAP were observed. After the personal intervention ended the values remained below the preintervention period (40.9% in the fourth quarter of 2019 and 54.3% in the first quarter of 2020; Figure 4).

### Duration of antibiotic therapy and prophylaxis

In the preintervention period, the median duration of antibiotic therapy was 12 days (range 5–28d, Table 1). This figure dropped to 8 days (range 3–46 d) at the end of the personal intervention and rose to 8.5 and 9.5 days (range 2–41 and 2–18 d) in the consecutive quarters after the intervention respectively. For the duration of the prolonged SAP an even more marked effect was seen: The median duration before the AMS intervention was 9 days (range 2–22 d), 4 days (range 1–11 d) at the end of the personal intervention and dropped further to 2 days (range 1–17 d) in the postintervention period.

**Table 1.** Duration of antibiotic therapy and prophylaxis at the Department of Oral and Maxillofacial Surgery

Period	Yearly quarter	Duration of therapy [days]	Range [days]	Duration of prophylaxis [days]	Range [days]
Preintervention	III. 2018	12,0	5;28	9,0	2;22
Intervention	IV. 2018	10,0	2;33	4,0	1;44
	I. 2019	8,0	3;28	3,0	1;10
	II. 2019	6,5	4;21	5,0	1;9
	III. 2019	8,0	3;46	4,0	1;11
Postintervention	IV. 2019	9,5	2;41	3,0	2;16
	I. 2020	8,5	2;18	2,0	1;17

### Antibiotic substances

During the overall study period, beta-lactam/ beta-lactamase inhibitor combinations were the predominant antibiotic class; 75.6% of all patients received Ampicillin-Sulbactam. The second most commonly used antibiotic was Clindamycin which was given to 11.6% of all patients 70% of which had a penicillin allergy label. Metronidazol was administered to 7% of all patients. The use of restricted antibiotics was rare: Fluoquinolones 3.3%; Vancomycin, Daptomycin, Linezolid combined: 1.7%; Meropenem 0.6%. In total, 11.2% of all patients received more than one antibiotic. None of these proportions changed significantly during or after the intervention period.

### Discussion

This study demonstrates a significant and sustained reduction in antibiotic consumption within an OMFS department following the implementation of an Antimicrobial Stewardship AMS intervention. It shows the overall effectiveness of a multifaceted stewardship approach that included audit and feedback, guideline implementation, and educational efforts.

There are no available comparable data regarding the prevalence of antibiotic use specifically in maxillofacial surgical patients. However, in a German nationwide prevalence study from 2016, an overall prevalence of antibiotic use of 30.1% was found on surgical non-intensive care wards.^11^ In comparison, at our OMFS ward, the prevalence of patients that received any antibiotic during the preintervention period was 72% which seems extraordinarily high.

Over the entire study period, antibiotic use was reduced by approximately 26%, which is a substantial decline and all the more remarkable as the opposite trend was observed in the comparator group, consisting of OMFS wards from other university hospitals with presumably comparable patient demographics and clinical conditions. Here, an increase in antibiotic consumption of almost 50% during the same time period was reported.

Importantly, the initial phase of the intervention—the so-called “handshake stewardship,” involving weekly interdisciplinary ward visits with direct discussions and written recommendations—alone resulted in substantial changes in prescribing behavior. Even before the formal implementation of the consensus guideline, a marked decrease in both the prevalence of antibiotic use and the duration of therapy was observed. This highlights the power of personal interaction, regular feedback, and shared decision-making in modifying clinical practice.

Another important observation was the high proportion of patients receiving prolonged SAP. In the published literature, usually 15–17% of antibiotics are allocated to SAP,^12,13^ however deviation from published protocols are common in different countries and settings.^14^ ^–^ ^16^ In our cohort, 68.6% of patients received antibiotics in the context of prolonged postoperative prophylaxis beyond the generally recommended single shot regimens given directly before an operation as these were administered in preop theater and thus not documented in our cohort.

The indication of SAP in OMFS is complex. OMFS encompasses an exceptionally broad spectrum of procedures, ranging from skin and dentoalveolar surgery to the management of facial trauma, orthognathic interventions, cleft lip and palate repair, and tumor surgery. This diversity of operative fields makes the specialty unique among surgical disciplines, as it integrates functional, reconstructive, and aesthetic aspects within a single domain.^17^ While perioperative antibiotic prophylaxis is often justified in high-risk surgeries, such as extensive tumor resections with flap reconstruction, the absence of robust evidence and standardized national guidance frequently leads to overly broad and prolonged use. OMF surgery further differs from other surgical specialties in that aspect that often naturally highly contaminated wound areas as the oral cavity are involved. Given the often non sterile surgical site as well as additional patient associated risk factors like insufficient oral hygiene, diabetes mellitus, former radiation or osteonecrosis, wound contamination wound infections are intuitively predictable and thus feared.^18,19^

However, under the umbrella of patient safety, and denial of the harmful effects of prolonged antibiotic administration inappropriate SAP and antibiotic therapy exposes patients to additional risks as *C. difficile* infection and accelerated antimicrobial resistance selection.^20,21^ This underscores the importance of critically reassessing prophylactic practices and tailoring them to specific risk constellations and procedures. At the time of the intervention, no national guideline addressing the majority of OMFS procedures had been published. After a literature review for all procedures requiring SAP, we agreed on a single shot prophylaxis with the exception of advanced flap procedures and surgical interventions associated with medication-related osteonecrosis of the jaw.

One of the most encouraging effects of our intervention was the reduction in the antibiotics used for SAP. In the preintervention period, nearly 70% of antibiotics were used as postsurgical prophylaxis with a median duration of 9 days, exceeding any guideline-recommendations. During and after the intervention, this dropped substantially, with prophylactic use representing only 40–54% of prescriptions in later quarters. Notably, the duration of SAP decreased to 3 days and declined further to 2 days after the intervention stopped. In a parallel study, we assessed whether the reduction of SAP to a single shot regimen had an influence on outcome and found that reduced-duration antibiotic prophylaxis was not associated with increased rates of postoperative complications as wound dehiscence or clinical infection but reduced the length of stay for several oral and maxillofacial procedures.^22^ The reduction seen due to the AMS intervention indicates increased awareness and critical evaluation of prophylactic indications, particularly important in a field like OMFS. Given the high values for antibiotic consumption from the comparator hospitals, is seems likely that the high rate and duration of prophylaxis is not a local but general phenomenon. In the meantime, several studies have confirmed our approach and demonstrated that single-shot prophylaxis does not lead to an increased rate of postoperative wound infections in many surgical procedures.^23^ ^–^ ^26^ However, further studies are warranted that confirm the safety of shortened SAP regimens in certain OMFS procedures.

Encouragingly, the reduction in antibiotic consumption was sustained even after the end of the intensive, personal AMS intervention. Although some rebound was observed in subsequent years, antibiotic use remained below both preintervention levels and the national average, suggesting a sustainable change within the department. This durability is likely due to the combination of behavioral change and written recommendations. Frequent personal interactions during the intervention forstered a trustful relationship between OMFS surgeons and the AMS team, who very perceived as a supportive and resourceful clinical partner rather than antibiotic gatekeepers. This collegial relationship extended beyond the intervention period, with the AMS team regularly consulted in complex clinical cases. In addition, the introduction of clear, mandatory consensus-based guidelines provided structure and continuity after the direct oversight ended, supporting especially young clinicians or less experienced staff not yet fully familiar with local procedures.

In comparison with other studies in surgical disciplines, this intervention achieved comparable or even greater reductions in antibiotic consumption. Similar AMS strategies have shown reductions ranging from 20–30%, particularly when combining personal interaction with structural tools like guidelines.^27–29^ One strength of our study is its long observation period, allowing the assessment of sustained effects beyond the active intervention. However, this study has several limitations. It only includes data from a single-center and due to its prepost design, the absence of a control group and possible undetected temporal confounders or secular trends in antibiotic use limit the ability to attribute observed effects solely to the intervention. The limited patient numbers and heterogeneity of clinical presentations and surgical interventions made it impossible to stratify the results according to different risk groups and precluded robust statistical analyses. Most importantly, data on this specific cohort regarding clinical outcomes are lacking, which would further validate the safety of the overall reduced antibiotic use. Also, we did not assess possible side effects of antibiotic use. However, in a different study covering patients over two years from 2018–2020, we could show that at least the reduction in the SAP to a single shot regimen was not associated with increased postoperative complications.^22^ It is reasonable to assume that the marked reduction in antibiotic use, prevalence and duration of antibiotic prophylaxis reflect a genuine and clinically relevant effect of the intervention.

Overall, this study supports the effectiveness and sustained impact of structured Antimicrobial Stewardship (AMS) interventions in surgical settings and emphasizes the particular importance of addressing prophylactic antibiotic use in OMFS. Our findings underscore that personal engagement—like “handshake stewardship” through regular ward visits and face-to-face consultations—is key in changing prescribing behavior. Well-resourced and adequately funded AMS teams are therefore essential for improving antibiotic therapy in surgical care.

## References

[1] Vila PM , Zenga J , Jackson RS. Antibiotic prophylaxis in clean-contaminated head and neck surgery: a systematic review and meta-analysis. Otolaryngol Head Neck Surg 2017;157:580–588. 28695786 10.1177/0194599817712215

[2] Lauder A , Jalisi S , Spiegel J , Stram J , Devaiah A. Antibiotic prophylaxis in the management of complex midface and frontal sinus trauma. The Laryngoscope 2010;120:1940–1945. 20824781 10.1002/lary.21081

[3] Bratzler DW , Dellinger EP , Olsen KM , Perl TM , Auwaerter PG , Bolon MK. Clinical practice guidelines for antimicrobial prophylaxis in surgery. Surg Infect 2013;14:73–156. 10.1089/sur.2013.9999 23461695

[4] Schuderer JG , Hoferer F , Eichberger J , et al. Predictors for prolonged and escalated perioperative antibiotic therapy after microvascular head and neck reconstruction: a comprehensive analysis of 446 cases. Head Face Med 2024;20:58. 39402552 10.1186/s13005-024-00463-9 PMC11475970

[5] Mitchell RM , Mendez E , Schmitt NC , Bhrany AD , Futran ND. Antibiotic prophylaxis in patients undergoing head and neck free flap reconstruction. JAMA Otolaryngol Head Neck Surg 2015;141:1096–1103. 25905902 10.1001/jamaoto.2015.0513

[6] Habib AM , Wong AD , Schreiner GC , et al. Postoperative prophylactic antibiotics for facial fractures: a systematic review and meta-analysis. The Laryngoscope 2019;129:82–95. 29756330 10.1002/lary.27210

[7] Sandu AM , Chifiriuc MC , Vrancianu CO , et al. Healthcare-associated infections: the role of microbial and environmental factors in infection control-A Narrative Review. Infect Dis Ther 2025;14:933–971. 40208412 10.1007/s40121-025-01143-0 PMC12084486

[8] GBD 2021 Antimicrobial Resistance Collaborators. Global burden of bacterial antimicrobial resistance 1990–2021: a systematic analysis with forecasts to 2050. Lancet 2024;404:1199–1226. 39299261 10.1016/S0140-6736(24)01867-1 PMC11718157

[9] Barlam TF , Cosgrove SE , Abbo LM , et al. Implementing an antibiotic stewardship program: guidelines by the infectious diseases society of America and the society for healthcare epidemiology of America. Clin Infect Dis 2016;62:e51–77. 27080992 10.1093/cid/ciw118 PMC5006285

[10] Methodology WCCfDS. Guidelines for ATC classification and DDD assignment 2024. Oslo: WHO Collaborating Centre for Drug Statistics Methodology. 2024. https://atcddd.fhi.no/filearchive/publications/2024_guidelines__final_web.pdf. Accessed October 1, 2025.

[11] Aghdassi SJS , Gastmeier P , Piening BC , et al. Antimicrobial usage in German acute care hospitals: results of the third national point prevalence survey and comparison with previous national point prevalence surveys. J Antimicrob Chemother 2018;73:1077–1083. 29309607 10.1093/jac/dkx494

[12] Sartelli M , Coccolini F , Labricciosa FM , et al. Surgical antibiotic prophylaxis: a proposal for a global evidence-based bundle. Antibiotics (Basel) 2024;13:100. 38275329 10.3390/antibiotics13010100 PMC10812782

[13] Behnke M , Aghdassi SJ , Hansen S , Diaz LAP , Gastmeier P , Piening B. The prevalence of nosocomial infection and antibiotic use in German hospitals. Dtsch Arztebl Int 2017;114:851–857. 29271343 10.3238/arztebl.2017.0851 PMC5762998

[14] Kıymaz Y , Karakök T , Büyükkörük M , et al. Evaluation of surgical antimicrobial prophylaxis compliance: a multicenter point prevalence study. Am J Infect Control 2025;53:552–558. 39855272 10.1016/j.ajic.2025.01.014

[15] Schmitt C , Lacerda RA , Turrini RNT , Padoveze MC. Improving compliance with surgical antibiotic prophylaxis guidelines: a multicenter evaluation. Am J Infect Control 2017;45:1111–1115. 28629754 10.1016/j.ajic.2017.05.004

[16] Bull AL , Worth LJ , Spelman T , Richards MJ. Antibiotic prescribing practices for prevention of surgical site infections in Australia: increased uptake of national guidelines after surveillance and reporting and impact on infection rates. Surg Infect 2017;18:834–40. 10.1089/sur.2017.119 28885898

[17] PE MMGGLPW. Peterson’s Principles of Oral and Maxillofacial Surgery. 4th ed: Springer; 2016. 2350.

[18] Campisi G , Mauceri R , Bertoldo F , et al. Medication-related osteonecrosis of jaws (MRONJ) prevention and diagnosis: Italian consensus update 2020. Int J Environ Res Public Health 2020;17: 5998. 32824826 10.3390/ijerph17165998 PMC7460511

[19] Martin ET , Kaye KS , Knott C , et al. Diabetes and risk of surgical site infection: a systematic review and meta-analysis. Infect Control Hosp Epidemiol 2016;37:88–99. 26503187 10.1017/ice.2015.249 PMC4914132

[20] Worldwide Antimicrobial Resistance National/International Network Group (WARNING) Collaborators . Ten golden rules for optimal antibiotic use in hospital settings: the WARNING call to action. World J Emerg Surg 2023;18:50. 37845673 10.1186/s13017-023-00518-3 PMC10580644

[21] Branch-Elliman W , O’Brien W , Strymish J , Itani K , Wyatt C , Gupta K. Association of duration and type of surgical prophylaxis with antimicrobial-associated adverse events. JAMA Surg 2019;154:590–598. 31017647 10.1001/jamasurg.2019.0569 PMC6487902

[22] Schorn L , Singh DD , Mrochen F , et al. Is single-shot antibiotic prophylaxis really enough for standard OMF-surgeries? Clin Oral Investig 2026;30:68. 10.1007/s00784-026-06756-4 PMC12861997 41622382

[23] Van der Cruyssen F , Forrest M , Holmes S , Bhatti N. A systematic review and meta-analysis of fracture-related infections in maxillofacial trauma: incidence, risk factors, and management strategies. J Clin Med 2025;14:1332. 40004862 10.3390/jcm14041332 PMC11856410

[24] Santamaría Arrieta G , Rodríguez Sánchez F , Rodriguez-Andrés C , Barbier L , Arteagoitia I. The effect of preoperative clindamycin in reducing early oral implant failure: a randomised placebo-controlled clinical trial. Clin Oral Investig 2023;27:1113–1122. 10.1007/s00784-022-04701-9 PMC9469834 36098814

[25] Remschmidt B , Schwaiger M , Gaessler J , Wallner J , Zemann W , Schwaiger M. Surgical site infections in orthognathic surgery: prolonged versus single-dose antibiotic prophylaxis. Int J Oral Maxillofac Surg 2023;52:219–226. 35760661 10.1016/j.ijom.2022.06.002

[26] Kluba S , Reinert S , Krimmel M. Single-dose versus prolonged antibiotic prophylaxis for alveolar bone grafting in cleft patients. Int J Oral Maxillofac Surg 2023;52:564–568. 36274022 10.1016/j.ijom.2022.10.002

[27] Yeo CL , Chan DS , Earnest A , et al. Prospective audit and feedback on antibiotic prescription in an adult hematology-oncology unit in Singapore. Eur J Clin Microbiol Infect Dis 2012;31:583–590. 21845470 10.1007/s10096-011-1351-6

[28] Jenkins TC , Knepper BC , Sabel AL , et al. Decreased antibiotic utilization after implementation of a guideline for inpatient cellulitis and cutaneous abscess. Arch Intern Med 2011;171:1072–1079. 21357799 10.1001/archinternmed.2011.29

[29] Carling P , Fung T , Killion A , Terrin N , Barza M. Favorable impact of a multidisciplinary antibiotic management program conducted during 7 years. Infect Control Hosp Epidemiol 2003;24:699–706. 14510254 10.1086/502278

